# Improved Glucocorticoid Receptor Ligands: Fantastic Beasts, but How to Find Them?

**DOI:** 10.3389/fendo.2020.559673

**Published:** 2020-09-24

**Authors:** Laura Van Moortel, Kris Gevaert, Karolien De Bosscher

**Affiliations:** ^1^Translational Nuclear Receptor Research (TNRR) Laboratory, VIB, Ghent, Belgium; ^2^VIB Center for Medical Biotechnology, VIB, Ghent, Belgium; ^3^Department of Biomolecular Medicine, Ghent University, Ghent, Belgium

**Keywords:** glucocorticoids, glucocorticoid receptor, selective GR modulators, drug discovery, inflammation, assay development, GR, SEGRM

## Abstract

Exogenous glucocorticoids are widely used in the clinic for the treatment of inflammatory disorders and hematological cancers. Unfortunately, their use is associated with debilitating side effects, including hyperglycemia, osteoporosis, mood swings, and weight gain. Despite the continued efforts of pharma as well as academia, the search for so-called selective glucocorticoid receptor modulators (SEGRMs), compounds with strong anti-inflammatory or anti-cancer properties but a reduced number or level of side effects, has had limited success so far. Although monoclonal antibody therapies have been successfully introduced for the treatment of certain disorders (such as anti-TNF for rheumatoid arthritis), glucocorticoids remain the first-in-line option for many other chronic diseases including asthma, multiple sclerosis, and multiple myeloma. This perspective offers our opinion on why a continued search for SEGRMs remains highly relevant in an era where small molecules are sometimes unrightfully considered old-fashioned. Besides a discussion on which bottlenecks and pitfalls might have been overlooked in the past, we elaborate on potential solutions and recent developments that may push future research in the right direction.

## Introduction

**Figure 1:**
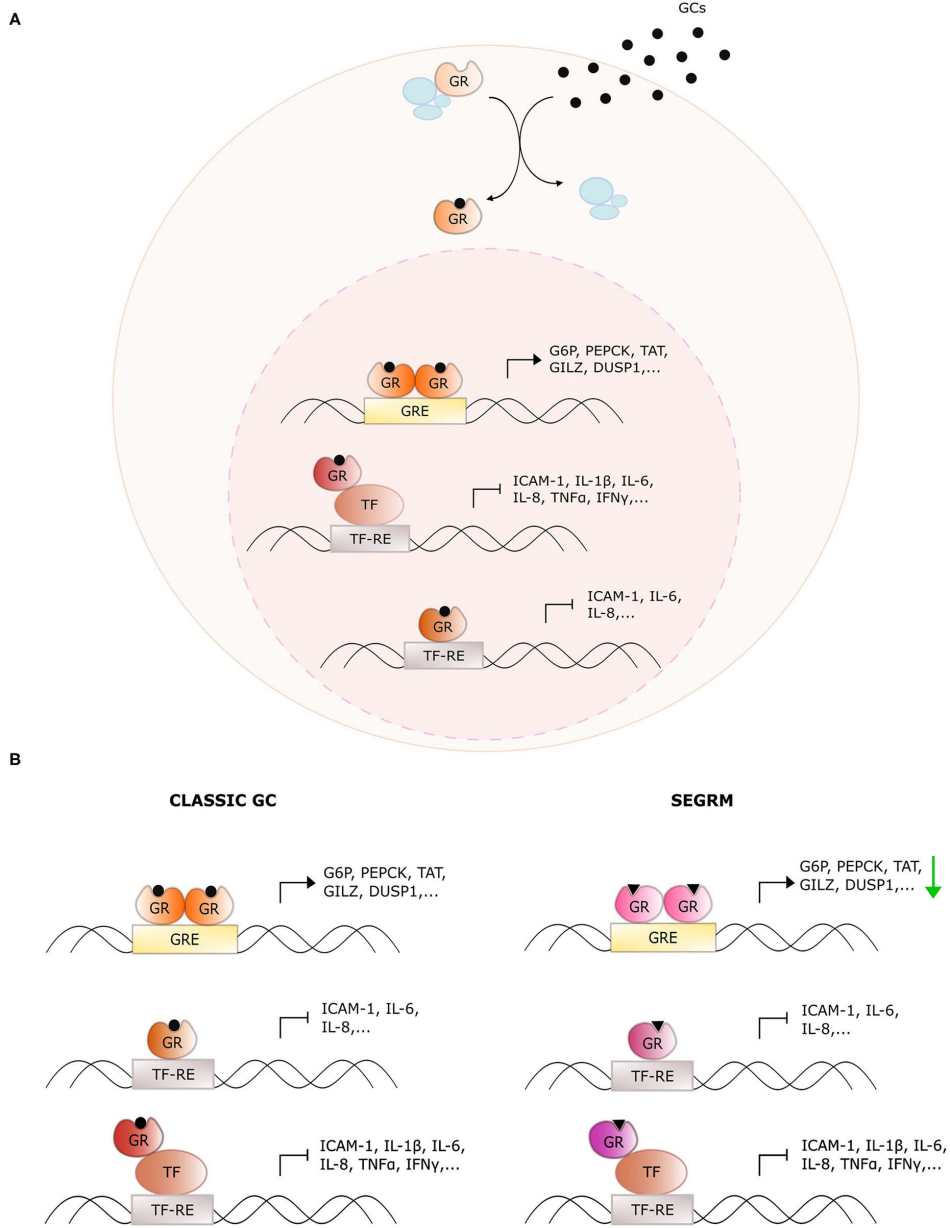
Overview of glucocorticoid receptor activity with classic glucocorticoids and selective GR modulators. **(A)** General action mechanism of the glucocorticoid receptor (GR). Glucocorticoids (GCs) diffuse through the cellular membrane and bind GR. The latter dissociates from its chaperone complex and migrates to the nucleus. There, it dimerizes and binds glucocorticoid response elements (GREs) to upregulate downstream target genes. Monomeric GR also undergoes protein-protein interactions with DNA-bound pro-inflammatory transcription factors (TFs) to downregulate their activity, or it binds directly to the TF response elements (TF-RE). **(B)** Distinct actions of classic GCs and selective GR modulators (SEGRMs). In contrast to classic GCs, SEGRMs are hypothesized to reduce GR's capacity to dimerize and therefore reduce GRE-mediated transcription. Interference with TF activity is driven via monomeric GR and therefore maintained with SEGRMs.

Glucocorticoids (GCs) are endogenous steroidal hormones involved in metabolism, stress, development, and immunity (1). They exert their effects by binding the glucocorticoid receptor (GR), a nuclear receptor (NR) consisting of an intrinsically disordered N-terminal domain (NTD), a central DNA binding domain (DBD), a hinge region (HR), and a C-terminal ligand-binding domain (LBD) (2). Upon ligand binding, GR typically translocates from the cytoplasm to the nucleus where it acts as a genuine transcription factor to regulate target gene expression via multiple mechanisms (Figure 1A), which are discussed in detail in (3). The discovery of the anti-inflammatory effects of endogenous GCs preceded the development of synthetic GCs, which are used to treat, among others, inflammatory disorders, and hematological cancers (4). Unfortunately, the therapeutic efficacy of such exogenous GCs is, particularly for systemic use, overshadowed by an unacceptably high number of undesired side effects such as hyperglycemia, osteoporosis, mood swings, and weight gain (5).

Some of these side effects stem from direct binding of homodimeric GR to pseudopalindromic glucocorticoid reponse elements (GREs) in the promoter regions of genes controlling key metabolic pathways (Figure 1). The resulting GRE-driven upregulation of tyrosine aminotransferase (TAT), glucose 6-phosphatase (G6P) and phosphoenolpyruvate carboxykinase (PEPCK) for instance leads to hyperglycemia (6). The suppression of nuclear factor (NF)-κB- and activator protein (AP)-1 activity on the other hand, is typically explained via protein-protein interactions with monomeric GR (called tethering) (7). Despite the controversies on the actual underlying mechanism (see further), the targeting of activities of pro-inflammatory transcription factors undoubtedly contributes substantially to the anti-inflammatory actions of GCs.

The discrepancy between monomer- and dimer-driven effects of GR was first suggested in 1994 with the demonstration that GR with a dimerization-disrupting mutation in the DBD (GRdim) is still able to repress AP-1-driven genes, while no longer able to induce GRE-mediated activation (8). Four years later, Reichardt et al. established that mice carrying this homozygous mutation were viable and healthy, in contrast to GR full knock-out mice (9), arguing for an equally viable mechanistic basis to separate beneficial from undesired effects. This was the starting point of the search for so-called dissociative or selective GR modulators (SEGRMs), GR ligands that can still repress inflammation via monomer-driven NF-κB, and AP-1 inhibition, while no longer inducing GRE-driven side effects (Figure 1B).

In the meantime, a few shades of gray have been added to the original black-and-white monomer-dimer paradigm. First of all, dimer-mediated gene activation also contributes to the anti-inflammatory effects of GCs via the upregulation of anti-inflammatory genes such as glucocorticoid-induced leucine zipper (GILZ) and dual specificity phosphatase (DUSP1) (10). This helps explaining why GRdim mice show increased sensitivity to acute inflammation such as septic shock (11). Secondly, it was shown *in cellulo* that Dex still promotes dimerization of the GRdim mutant (12). However, introducing an extra point mutation in the GR LBD almost completely disrupted dimerization and abrogated GRE-driven activity, but preserved the inhibition of NF-κB activity. Thirdly, monomeric GR was shown to bind directly to genomic NF-κB and AP-1 response elements, without the need for the transcription factor itself (13, 14). This finding challenges the original tethering hypothesis but still supports the notion that suppression of NF-κB and AP-1 activities does not require GR dimerization. Taken together, given the bodies of evidence on a large contribution of dimeric GR to particular side effects vs. the role of GR monomers to support anti-inflammatory actions in a chronic setting, the notion that compounds that favor signaling via monomeric GR can hold a therapeutic benefit against persistent inflammation, still stands.

The development of successful SEGRMs has proven to be a long and extremely bumpy road. Many compounds that showed promising initial results (listed in Table 1) never got past the pre-clinical stage or failed later on in clinical trials. It is well-known that the success rate for the development of any kind of small molecule drug from bench to clinic is very low, typically starting from 10,000 compounds to end up with one market-approved drug (44, 45). In addition, we believe that in the case of GR, multiple technical, and biological factors have been reducing the prospect to success even further. Fortunately, molecules are still being developed, trying to meet the hope of many patients who would benefit from GR modulators. For instance, AZD7495 (asthma, NCT03622112) and AZD9567 (rheumatoid arthritis, NCT03368235) are currently under evaluation in clinical trials.

**Table 1:** Available pre-clinical data of SEGRMs.

**Compound**	***In vitro* assays and *in cellulo* overexpression assays**	***In cellulo* assays for endogenous anti-inflammatory and/or side effect targets**	**Inflammatory animal models**	**Status and latest progress**	**References**
LGD-5552	Ligand-binding assays GR, AR, MR, PR MMTV-luciferase in CV-1 cells (overexpressed GR) E-selectin-luciferase in CV-1 and HepG2 cells (overexpressed GR) IL-6-luciferase in HepG2 cells (overexpressed GR) Cofactor binding assays	PEPCK and PDK4 mRNA in H4IIE cells COX2 and APOCIII mRNA in H4IIE cells POMC mRNA in ATT20 cells	Collagen-induced arthritis in mice Freund's complete adjuvant-induced arthritis in rats Experimental autoimmune encephalomyelitis in rats	Discontinued (preclinical)	(15, 16)
AL-438	Ligand-binding assays GR, PR RSV-LTR-GRE-luciferase in CV-1 cells (overexpressed GR) TAT-luciferase in HepG2 cells (overexpressed GR) E-selectin-luciferase in HepG2 cells (overexpressed GR) Cofactor binding assays	Eosinophil counts in BAL Human PBMC cell and rat splenocyte T-cell proliferation assays Osteocalcin protein in MG-63 cells Aromatase activity in hDSF cells IL-6 release in HSKF1501 cells	Carrageen-induced paw edema in rats Freund's complete adjuvant-induced arthritis in rats	Discontinued (preclinical)	(17–19)
MK-5932	MMTV-luciferase in A549 cells MMTV-luciferase in HeLa cells TNFα-β-lactamase in U937 cells	TNFα, IFNγ, IL-1β, IL-6 secretion in human whole blood TNFα, IL-6 secretion in rat whole blood	Oxazolone-induced contact dermatitis in rats	Discontinued (preclinical)	(20, 21)
GW870086	Functional selectivity MR, AR, PR, ER on MMTV-luciferase in CV-1 cells MMTV-LTR-luciferase in A549 and MG-63 cells E-selectin-κB-RE-alkaline phosphatase in A549 cells	Lymphotoxin-β, COX-2, Cyp24a1, MAP-7, GPR64, GILZ, DUSP1, MICAL2, FKBP5, CDKN1C, RGS2, SGK mRNA in A549 cells	Delayed-type oxazolone-induced contact hypersensitivity in mice Ovalbumin-induced airway inflammation in mice	Discontinued Phase II for asthma: no difference with placebo (NCT00945932) Phase II for atopic dermatitis: weaker effects than fluticasone propionate standard (NCT01299610)	(22–24)
BI653048	Ligand-binding assays GR, PR MMTV-luciferase in HeLa cells	IL-6 release in CCD-1112Sk cells Osteocalcin levels in MG-63 cells Human ERG potassium channel inhibition in Hek293T cells	Canine low dose endotoxemia model	Discontinued Phase I: no improvement on side effect profile compared to prednisolone (NCT02217631, NCT02224105, NCT02217644)	(25–27)
Mapracorat	Ligand-binding assays GR, PR, AR, MR MMTV-luciferase in HeLa cells Collagenase-luciferase in HeLa cells κB-RE-luciferase in SV-40 transformed hCEpiC cells TPA-RE-luciferase in SV-40 transformed hCEpiC cells	TAT activity in HepG2 cells IL-12p40, IFNγ secretion in PBMC cells Eotaxin-1 (+/– GR siRNA), −3, CCL5 (+/– GR siRNA), G-CSF, IL-6, IL-8, MCP-1 release in hConF cells Eotaxin-3, CCL5 (+/– GR siRNA), CCL27, ICAM-1 (+/– GR siRNA), IL-6, IL-8, MCP-1, TNFα release in hCEpiC cells IL-6, MCP-1 release and (p)p38, (p)JNK protein in SV-40 transformed hCEpiC cells IL-6, IL-8 release in hONA cells IL-1β, ICAM-1 release in hREC cells IL-6, IL-12p40, MCP-1 release in THP-1 cells (p)JNK, (p)p65, (p)p38, IκBα levels in hCEpiC cells MYOC levels in mkTM cells Migration, apoptosis, IL-8 release, annexin-1, and CXCR4 expression in human eosinophils IL-6, IL-8, CCL5, TNFα release in hMC-1 cells GM-CSF, TNFα, PGE2 production and COX-2, (p)p38, (p)MK2, DUSP1 protein in Raw 264.7 cells IL-6, IL-8, MCP-1, PGE2 release, COX-2, RelB, (p)IκBα protein, RelA and RelB DNA binding in human keratinocytes	Croton oil-induced irritant contact dermatitis in mice and rats Dinitrofluorobenzene (DNFB)-induced allergic contact dermatitis in mice and rats Dry eye model in rabbits Paracentesis model in rabbits Ovalbumin-induced allergic conjunctivitis in guinea pigs Compound 48/80-induced wheal and erythema skin inflammation in beagles	Discontinued Phase III for cataract surgery, no results reported (NCT01591655) Phase I for psoriasis, no results reported (NCT03399526) Phase I to assess corneal endothelial cell changes, no results reported (NCT01736462)	(28–36)
(Fos)dagrocorat	Gal4-RE-luciferase with Gal4-DBD-LBD in Huh7 cells Cofactor binding assays	IL-6 release in A549 cells IFNγ in human whole blood assays Human pre-adipocyte differentiation FABP4 mRNA in adipocytes TAT, PEPCK in human primary adipocytes Osteocalcin levels in human primary osteoblasts	Murine LPS-induced endotoxemia model	Discontinued Phase II for rheumatoid arthritis: no improved benefit-risk ratio compared to prednisone (NCT01393639)	(37, 38)
AZD5423	Ligand-binding assays GR, MR, PR, AR, ERα, ERβ TPA-RE-β-galactosidase stable in ChaGoK1 cells	TNFα-release in hPBMC cells	Sephadex-induced airway inflammation in rats	Discontinued Phase II for asthma (NCT01225549) Phase II for COPD (NCT01555099)	(39–41)
AZD7594	Ligand-binding assays GR, MR, PR, AR, ERα, ERβ TPA-RE-β-galactosidase stable in ChaGoK1 cells	TNFα-release in hPBMC cells	Sephadex-induced airway inflammation in rats	Ongoing Second phase II for asthma completed 11/2019 (NCT03622112) Phase I in adolescents ongoing (NCT03976869)	(39, 42)
AZD9567	Ligand-binding assays GR, MR, PR, AR, ERα, ERβ MMTV-β-galactosidase stable in ChaGoK1 cells TPA-RE-β-galactosidase stable in ChaGoK1 cells Cofactor binding assays	TAT in primary hepatocytes Osteoprotegerine in human fetal osteoblasts	Streptococcal cell wall reactivation arthritis model in rats	Ongoing Phase II for rheumatoid arthritis completed 11/2019 (NCT03368235)	(43)

*Information on clinical trials was retrieved from clinicaltrials.gov. A549, human lung epithelial carcinoma cell line; APOCIII, apolipoprotein C18; AR, androgen receptor; ATT20, mouse pituitary tumor cell line; BAL, bronchoalveolar lavage; CCD-1112Sk, human foreskin fibroblast cell line; CCL (5), C-C motif chemokine (5); CDKN1C, cyclin-dependent kinase inhibitor 1C; hCEpiC, human corneal epithelial cells; ChaGoK1, human bronchogenic carcinoma cell line; hConF, human conjunctival fibroblasts; COX-2, cyclo-oxygenase 2; CV-1, African green monkey kidney cell line; Cyp24a1, vitamin D (3) 24-hydroxylase; CXCR4, C-X-C chemokine receptor type 4; hDSF, human dermal skin fibroblasts; ER, estrogen receptor; hERG, human ether-a-go-go potassium channel; FKBP5, 51 kDa FK506-binding protein; G(M)-CSF, granulocyte (macrophage) colony-stimulating factor; GPR-64, G-protein coupled receptor 64; H4IIE, rat hepatocellular carcinoma cell line; Hek293T, human embryonic kidney cell line; HeLa, human cervical adenocarcinoma cell line; HepG2, human hepatocellular carcinoma cell line; HSKF1501, human foreskin fibroblast cell line; Huh7, human hepatocellular carcinoma cell line; ICAM-1, intracellular adhesion molecule 1; IFNγ, interferon γ; IκBα, NF-kappa-B inhibitor α; IL(-12p40), interleukin (12 subunit p40); (p)JNK, (phospho-)c-Jun N-terminal kinase; κB-RE, NF-κB response element; LTR, long terminal repeat; MAP-7, microtubule-associated protein 7; hMC-1, human mast cell line; MCP-1, monocyte chemotactic protein 1; MG-63, human osteosarcoma cell line; MICAL2, molecule interacting with CasL protein 2; (p)MK-2, (phospho-)mitogen-activated protein kinase-activated protein kinase 2; MMTV, mouse mammary tumor virus; MR, mineralocorticoid receptor; MYOC, myocillin; hONA, human optic nerve astrocytes; (p)p38, (phospho-)mitogen activated protein kinase p38; (p)p65, (phospho-)nuclear factor kappa B subunit p65; PBMC, peripheral blood mononuclear cells; PDK4, pyruvate dehydrogenase kinase 4; PGE2, prostaglandin E2; POMC, pro-opiomelanocortin; PR, progesterone receptor; Raw264.7, mouse leukemia macrophage cell line; hREC, human retinal endothelial cells; RGS2, regulator of G-protein signaling 2; RSV, Rous sarcoma virus; SGK, serum/glucocorticoid regulated kinase; THP-1, human leukemic monocyte cell line; mkTM, monkey trabecular meshwork cells; TPA-RE, 12-O-Tetradecanoylphorbol-13-acetate response element; U937, human histiocytic lymphoma cell line*.

This perspective offers our opinion as molecular biologists on the rationale why a continued search for SEGRMs still makes sense and bears significant relevance. We offer our view on a number of bottlenecks and pitfalls that might have hampered research progress in the past and elaborate on which new developments and insights could help overcome these issues.

## SEGRMs: The Unmet Medical Need

The need for more selective GR ligands remains highly relevant. Although more targeted therapies have successfully been introduced, such as anti-tumor necrosis factor (TNF) for arthritic disorders and inflammatory bowel diseases (IBD), these therapies are not without limitations. For one, anti-TNF therapy has been associated with a 250% increase in the occurrence of tuberculosis (46). Furthermore, these therapies have been reported to trigger multiple sclerosis (MS) and other demyelinating conditions (47–50). This is in line with the reported disease worsening in patients with pre-existing MS in clinical trials for Lenercept and cA2, two types of anti-TNF therapy (51, 52). Beside such side effects, monoclonal therapies are generally very expensive, laying a huge burden on health care systems, which will only increase with aging populations in western countries. Their price also makes them unaffordable for most people in low income countries, which is particularly a problem for asthma, for which 80% of disease-related deaths occur in low to low-medium income countries (53).

GCs on the other hand are generally much cheaper and are still the first-line treatment for asthma, multiple sclerosis, and multiple myeloma among others (54–56). However, their side effects are a well-known problem and not necessarily limited to patients receiving oral or intravenous GCs. While topical skin treatments, especially with the newest generation glucocorticoids, impose a very low risk for systemic side effects (57–59), topical eye treatments, and inhaled GCs (IGCs) have both been associated with adrenal suppression (60, 61). This can lead to growth retardation in infants and children, who form a large cohort of the asthma patient population. The long-term use of high doses IGCs has also been associated with decreased bone mineral density in both children and adults (62–64). Although the benefits of ocular and IGCs usually outweigh the risks, patients would still benefit from GCs with lower risks for systemic side effects.

Taken together, the need for more selective GCs reaches further than systemic treatments and is also high for ocular and inhalation therapies.

## Bottlenecks and Pitfalls Observed in the Past

Current tools for screening potential SEGRMs suffer from shortcomings and do not always capture the complexity of GR signaling. First of all, the lack of three-dimensional structures of full-length GR highly restricts our knowledge of GR's structure-activity relationship and decreases the predictive power of molecular modeling and docking studies. Additionally, all existing crystal structures of ligands in complex with GR's LBD were obtained upon the introduction of one or more mutations in this LBD. Although these mutations were predicted not to influence the LBD structure, this can never be claimed with absolute certainty. F602S for instance, one of the most commonly used GR mutations allowing growth of LBD crystals, causes chemical shift perturbations in LBD nuclear magnetic resonance spectra compared to wild-type LBD (65). Furthermore, GR is allosterically regulated through interactions with its corresponding response elements and cofactors (66), and more general also for other NR members, conformational changes in one NR domain can allosterically alter the conformation of another domain within the same NR molecule (67). Thus, most probably the conformation of the LBD studied in isolation is an incorrect reflection of this domain's conformation in the full-length protein.

Further, while high affinity and selectivity for GR can be captured using *in vitro* ligand-binding assays, confirmations in a cellular or *in vivo* context are sometimes lacking. This harbors an inherent risk to miss out on off-target effects of the compound in question. Therefore, the confirmation of GR dependency in a cellular and an *in vivo* context is still an important validation to make, for instance by testing compounds in wild-type vs. GR knock-out models.

Table 1 provides an overview of the assays typically carried out to characterize GR-mediated actions of a set of well-known SEGRMs. To our opinion, a lack of predictive power is one of the problems most difficult to solve, especially when moving from simplified assays to more complex biology. Direct GRE-driven activity, potentially leading to side effects, is almost universally monitored via reporters driven by a mouse mammary tumor virus (MMTV) promoter. Although a fast and straightforward and thus defendable method for initial compound characterization, a GRE-driven reporter assay can be a poor predictor for regulation of endogenous GRE-driven genes, as was also observed for MMTV (15, 22). The effects of GCs are highly gene-specific and GRE-driven activity can differ depending on the sequence of the GRE and the surrounding chromatin environment (68–70). The use of overexpressed GR should also be avoided in such assays, as this may lead to compound potencies and efficacies that are not necessarily representative for an endogenous context. Additionally, not all side effects are dimer-driven and are therefore not predictable via GRE-driven reporters. Mimicking the right gene- and context-specificity of GR activity remains one of the greatest challenges. Making a switch from reporters driven by minimal recombinant promoters to more physiologically relevant promoters could already offer some benefit. These promoters would ideally belong to genes that are confirmed mediators of underlying therapeutic - or side effects. Validation on a well-representative set of relevant endogenous target genes is even more important (see below, section Potential Solutions: The Way Forward).

Cell- and tissue-specificity of GC actions is another variable parameter. The MMTV-driven reporter for instance showed stronger upregulation by GW870086 in bone osteosarcoma cells compared to lung epithelial carcinoma cells (22). It thus remains essential to screen compounds in cell types that are the best proxies for the underlying therapeutic and/or side effects *in vivo*, for instance the use of hepatocytes to study effects on glucose and lipid metabolism, or the use of osteoblast or osteoclast cell lines for drugs that would be used in arthritis patients. Further, although characterization of compound activity *in cellulo* is essential, this will always be an oversimplification of the situation in a living organism. Therefore, validation of an improved therapeutic benefit depends on representative animal models. While this is readily implemented for anti-inflammatory effect scoring, concomitant testing of side effect parameters (such as glucose tolerance, insulin tolerance, cortisol levels, bone mineral density) presents a bottleneck, because a longer treatment protocol may be needed to surpass the thresholds of measurable results for these parameters or because of species differences (see below) (22, 43).

Lack of translatability from animal models to human patients is yet another hurdle to overcome. Differences in ligand activity between species can be an underlying cause, as observed for AL-438 and MK-5932, which both had stronger anti-inflammatory effects in rat vs. human blood (17, 20). While it would be recommended to perform initial cellular tests in human cells as much as possible, *in vivo* interspecies differences remain a hurdle in the entire field of drug discovery and are currently difficult to overcome. Another concern is when animal models used to study a particular disease insufficiently mimic the pathology observed in humans. A careful design and set-up of animal models remains key to study anti-inflammatory as well as side effects. If a well-known side effect (marker) of a classic GC in man is not observed in the animal model used, this model will obviously have no predictive power on (markers of) this particular side effect in patients and will therefore be unsuited to evaluate the improved benefit-risk ratio of SEGRMs over classic GCs. For instance, in a canine model of low dose endotoxemia used to investigate the anti-inflammatory and bone-sparing effects of BI653048, neither BI653048 nor prednisolone treatment affected osteocalcin levels (25). However, prednisolone does reduce bone mineral density in dogs and decreases bone formation markers in humans after 1 day (71, 72). Indeed, in a phase I clinical trial, BI653048, and prednisolone both caused decreased serum osteocalcin levels (26). Studies with other SEGRMs also concluded that osteocalcin levels *in cellulo* do not always reflect *in vivo* decreases in bone density (18, 26, 27, 37, 38), casting doubts on the value of osteocalcin as proxy for the *in vivo* reduction of bone mineral density.

Lastly, notwithstanding the notion that dissociating GCs may improve the benefit-risk ratio in chronic inflammatory disorders, a portion of the anti-inflammatory effects of GR does remain dimer-driven (73). Hence, the likelihood decreases for truly dissociating compounds to match the therapeutic efficacy of the strongest classic GCs. Taking into account that some side effects, such as osteoporosis, are at least partially mediated by monomeric GR (18), makes the quest to find a SEGRM that scores better on multiple side effects even more challenging.

## Potential Solutions: The Way Forward

Although pre-clinical characterization of compounds will never suffice to accurately predict their effects in patients, particular improvements on current screenings could increase the predictive power. First of all, reporter genes driven by physiological promoters relevant for the clinical context of the tested SEGRM should be preferred over artificial promoters. An example could be the use of the G6P- or PEPCK-promoters in liver cells to monitor hyperglycemia (74, 75), or a Runt-related transcription factor (Runx)2-driven promoter in osteoblasts or Smad-driven promoters in osteoclasts as markers for GC-induced osteoporosis (76, 77). A consistent and thorough screening of endogenous targets in a relevant human cellular context adds to importance. While monitoring GR activity in every targeted pathway for every compound is impossible to achieve, identification of reliable *in cellulo* biomarkers with a higher predictive power for species-independent *in vivo* anti-inflammatory and/or side effects would be a tremendous help. This requires a full understanding of the molecular mechanisms driving both anti-inflammatory and side effects in human tissues as well as in animal models. This is, particularly for side effects, not always the case. Continued efforts to unravel the underlying molecular mechanisms driving particular GC side effects are therefore crucial. However, some important side effect mediators have already been identified and could be suitable markers. Examples are muscle ring finger (MuRF)1, atrogin-1, and Krüppel-like factor (KLF)15 in muscle atrophy (78), regulated in development and DNA damage response (REDD)1 in skin (and muscle) atrophy (79, 80), and G6P and PEPCK in liver (74, 75). In bone, the upregulation of cleaved caspase 3 and −9 or the reduction of bone morphogenetic protein (BMP)2 and Runx2 activity are important predictors for reduction in osteoblast numbers (81, 82), while upregulation of receptor activator of nuclear factor-κB ligand (RANKL)-RANK signaling and cathepsin K activity are important markers for increased osteoclast differentiation and activity (respectively) (76, 83).

Reduction of publication bias toward “negative results” and joining forces between pharmaceutical companies and academic groups should push the current boundaries and drive research forward. At times, underlying reasons for discontinuation of (pre-)clinical research remain enigmatic. As one concrete example of many other examples that can be brought forward, results from three completed phase III clinical trials on the use of Mapracorat for post-operative treatment of cataract surgery (NCT01230125, NCT01591161, and NCT01591655) await publication, leaving fundamental scientists on the sideline wondering why Mapracorat was never market approved. More insights on where exactly discontinued SEGRMs failed, if those reasons are on the scientific level, will encourage academic labs with the right expertise to dig deeper into the underlying causes, and create feedback-knowledge that may flow back to industrial programs.

Even though fully dissociating SEGRMs might never reach the therapeutic efficacy of the most potent classic GCs, they can still offer relevant therapeutic benefit. Many inflammatory disorders are characterized by a disease course that alternates between periods of remission and exacerbation or relapse. SEGRMs might not trigger the full-on anti-inflammatory cascade that is required to suppress an exacerbation, but might be ideal for maintenance therapy. To maintain disease control, lower GC doses often suffice. SEGRMs could match the anti-inflammatory efficacy of the lower dose classic GCs while still showing a reduced side effect burden. Combination of classic GCs with SEGRMs or other therapeutic agents is another strategy to increase benefit-risk ratios. Combination of Dex with CpdA was for instance shown to increase anti-inflammatory effects while reducing GRE-driven signaling *in cellulo* (84). Finally, the development of compounds that do not bind the classic ligand-binding pocket but instead target the dimerization interface might be an interesting alternative strategy to disrupt GRE-driven signaling.

The intrinsically disordered nature of the GR NTD (85), has so far prohibited resolving a crystal structure of full-length GR. However, some smaller (however technically still challenging) advances could already lead to important new insights. Crystal structures of wild-type LBD in absence of stabilizing mutations would already give more confidence in the reliability of current docking approaches. Secondly, crystal structures of the DBD-hinge-LBD portion would not only lead to a better understanding of the structure-activity relationship of GR, but might pose extra advantages for molecular modeling or docking studies. Since efficient GR dimerization seems to require both DBD and LBD (12), crystal structures of at least the DBD-hinge-LBD portion of GR should improve predictions on those molecular entities that are truly dimer-disrupting.

Another important emerging strategy to find more efficacious GCs is to minimize exposure to non-inflamed tissues. IGCs can for instance be optimized to undergo rapid elimination once they enter the systemic circulation, a strategy that was applied for the development of AZD7594 (39). For systemic GCs, the use of liposomal formulations is showing very promising results. While these not only improve distribution to tissues that are anatomically difficult to reach (86–88), they can lower the side effects of systemic GCs by maximizing concentrations at the inflamed tissues while minimizing distribution to other tissues (89, 90).

While it may be utopia to try and develop compounds that alleviate all side effects, improved profiles for particular side effects may be achievable. Skin thinning and ocular hypertension are for instance among the most problematic side effects for topical and ocular GC treatments, respectively (5). For systemic treatments, liposomal formulations in combination with selective improvement of particular side effects may be a viable way forward. Liposomal SEGRMs that do not affect bone metabolism might for instance have an increased benefit-risk ratio over classic GCs for the treatment of arthritic disorders.

## Conclusion

While there still seems a long road ahead toward SEGRMs with a real improved benefit-risk ratio, there is light at the end of the tunnel. The pipeline of SEGRM compounds under clinical evaluation is not empty and new insights from ongoing (or future) research is expected to lead to optimized screening tools with maximized predictive power. Additionally, strategies to limit exposure to off-targets tissues, such as liposomal formulations for systemic treatments, show promising results (86–90). Combination of these approaches with the identification of reliable markers to predict on-target side effects, (e.g., ocular hypertension in ocular treatment, osteoporosis in rheumatoid arthritis, skin thinning in topic applications) may be an effective and achievable leap forward.

## Data Availability Statement

The original contributions presented in the study are included in the article/supplementary material, further inquiries can be directed to the corresponding author/s.

## Author Contributions

LVM wrote the manuscript with contributions from KG and KDB. LVM made the artwork with contributions from KG and KDB. All authors approved the final version.

## Conflict of Interest

The authors declare that the research was conducted in the absence of any commercial or financial relationships that could be construed as a potential conflict of interest.
